# Physiological of biochar and α-Fe_2_O_3_ nanoparticles as amendments of Cd accumulation and toxicity toward muskmelon grown in pots

**DOI:** 10.1186/s12951-021-01187-7

**Published:** 2021-12-20

**Authors:** Yunqiang Wang, Zhengkang Zou, Xinliang Su, Fengting Wan, Ying Zhou, Zhen Lei, Licong Yi, Zhaoyi Dai, Junli Li

**Affiliations:** 1 Institute of Economic Crops, Hubei Academy of Agricultural Science, Wuhan, 430064 People’s Republic of China; 2 School of Chemistry, Chemical Engineering and Life Sciences, Wuhan University of Technology, Wuhan, 430070 People’s Republic of China; 3 Vegetable Germplasm Innovation and Genetic Improvement Key Laboratory of Hubei Province, Hubei Academy of Agricultural Sience, Wuhan, 430064 People’s Republic of China

**Keywords:** Cd, Muskmelon, Biochar, α-Fe_2_O_3_ NPs, Toxicity, Differentially expressed genes

## Abstract

**Background:**

Due to the severe cadmium (Cd) pollution of farmland soil, effective measures need to be taken to reduce the Cd content in agricultural products. In this study, we added α-Fe_2_O_3_ nanoparticles (NPs) and biochar into Cd-contaminated soil to investigate physiological responses of muskmelon in the whole life cycle.

**Results:**

The results showed that Cd caused adverse impacts on muskmelon (*Cucumis melo*) plants. For instance, the chlorophyll of muskmelon leaves in the Cd alone treatment was reduced by 8.07–32.34% in the four periods, relative to the control. The treatments with single amendment, α-Fe_2_O_3_ NPs or 1% biochar or 5% biochar, significantly reduced the soil available Cd content, but the co-exposure treatments (α-Fe_2_O_3_ NPs and biochar) had no impact on the soil available Cd content. All treatments could reduce the Cd content by 47.64–74.60% and increase the Fe content by 15.15–95.27% in fruits as compared to the Cd alone treatment. The KEGG enrichment results of different genes in different treatments indicated that single treatments could regulate genes related to anthocyanin biosynthesis, glutathione metabolism and MAPK signal transduction pathways to reduce the Cd toxicity.

**Conclusions:**

Overall the combination of biochar and α-Fe_2_O_3_ NPs can alleviate Cd toxicity in muskmelon. The present study could provide new insights into Cd remediation in soil using α-Fe_2_O_3_ NPs and biochar as amendments.

**Graphic Abstract:**

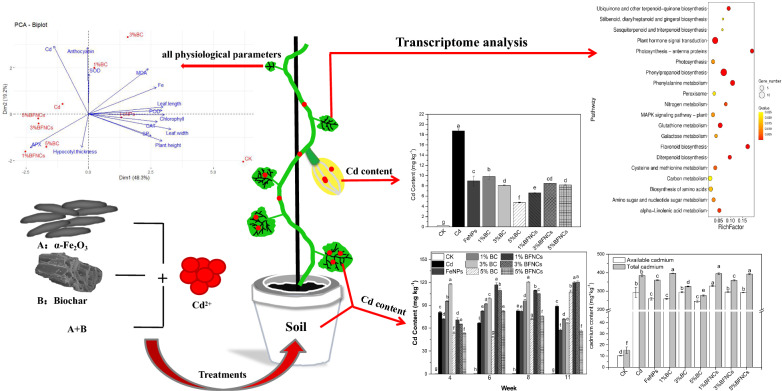

**Supplementary Information:**

The online version contains supplementary material available at 10.1186/s12951-021-01187-7.

## Synopsis

All the single or co-treatments could reduce the Cd content and increase the Fe content in fruit.

## Introduction

The industry development and human activities have made tremendous contributions to improving living standards, but they have also caused environmental problems, such as the accumulation of heavy metals in soil and water resources [1]. Nowadays, Heavy metal pollution continues to be one of the most serious environmental problems, which has attracted great attention [2]. In China, about 1/5 of the soil has been contaminated with heavy metals [3]. Cadmium (Cd) is an injurious heavy metal that has no biological importance [4]. Cd is extremely toxic to plants and can subsequently cause crop reduction in agriculture [5, 6]. In the soil, plants absorb Cd via roots, and these Cds eventually enter the human body through the food chain, which pose a great threat to human health [7]. For example, the prevalence of itai-itai disease in Japan was caused by Cd pollution [8]. Effective methods are urgently needed to remediate Cd contamination.

*In-situ* remediation technology is an effective method to reduce Cd pollution because of low cost and high efficiency [9]. Many remediating agents can be used to improve soil Cd pollution, such as Cellulose-zeolitic imidazolate frameworks (CelloZIFs), polylactic acid (PLA)-hydroxyapatite (HAp) composite, biochar, etc [10–12]. Biochar is a carbon-enriched material synthesized by pyrolysis of plant materials, which can be obtained from agricultural by-products [13]. Biochar has the characteristics of porous structure and high pH and because of its harmlessness to the environment and ability to improve crop productivity, it has attracted attentions in agricultural applications [14]. For example, biochar has great potential in reducing the effectiveness of soil Cd migration [13]. In addition, it was also demonstrated that biochar increased the biomass of Cd treated spinach by reducing the Cd accumulation in spinach [15]. Similarly, biochar reduced the Cd accumulation in super japonica rice and lettuce (*Lactuca sativa*) grown in Cd contaminated soil [16–18]. Therefore, biochar has considerable potential in remediating Cd contamination in soil.

Iron is an essential trace element for plant growth, which is closely related to the formation of chlorophyll in photosynthesis [19]. Due to their large surface area and high catalytic activity, nanomaterials have been widely used in industry and agriculture [20]. Fe_2_O_3_ nanoparticles (NPs) can be used as potential iron fertilizers in agriculture [21]. Recent studies have shown that iron oxide NPs also have a certain effect on remediating heavy metal polluted environments. For example, each gram of the hollow flower-like α-Fe_2_O_3_ nanostructures can adsorb 39 mg of Pb^2+^ or 48 mg of Cu^2+^ [22]. Even in comparison with traditional mercury adsorption methods, Fe_3_O_4_ NPs functionalized with gold showed a high adsorption affinity for mercury, with an adsorption capacity of 79.59 mg/g [23].

Magnetite (γ-Fe_2_O_3_) and hematite (α-Fe_2_O_3_) are natural forms of iron oxide in nature [24]. Previous studies have shown that α-Fe_2_O_3_ NPs have less negative effects on the growth and physiology of melon plants than γ-Fe_2_O_3_ NPs because of the excessive adsorption of γ-Fe_2_O_3_ NPs onto soil colloids with subsequent low water extractable iron [25, 26]. If α-Fe_2_O_3_ NPs can also reduce Cd pollution in the soil, and thus it can be used as an iron fertilizer or a remediating agent in the soil. It is reported that combining biochar and inorganic remediating agents such as zero valent iron and sulfur-based amendments may be better than individual applications [18]. However, effects of combination of Fe_2_O_3_ NPs and biochar on alleviating Cd-induced toxicity to terrestrial plants is still unclear.

Muskelon (*Cucumis melo*) is an important economic crop because its edible portion and seeds have medicinal values [27, 28]. The present experiment was conducted under greenhouse conditions to investigate the role of co-existing biochar and α-Fe_2_O_3_ NPs in altering Cd accumulation and toxicity to muskmelon plants, and further to optimize the combining ratio of the two amendments for soil remediation. The experiment was lasted until the maturity of muskmelon. At harvest, physiological and biochemical endpoints were measured across all treatments. Overall, these findings could provide important information for the restoration of soil Cd pollution and the safe cultivation of muskmelon plants on Cd-contaminated substrates.

## Materials and methods

### Biochar synthesis and material characterization

The soil is ordinary bagged matrix soil, and its physical and chemical properties are listed in Additional file 1: Table S1. α-Fe_2_O_3_ NPs (60 nm) were purchased from Macklin, Shanghai, China. Muskelon (*Cucumis melo*) seeds were obtained from Hubei Academy of Agricultural Sciences, China.

**Fig. 1:**
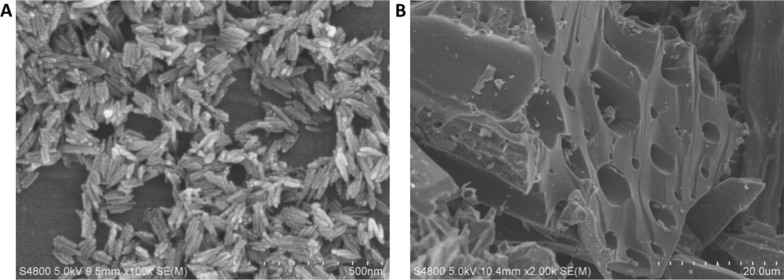
SEM images of α-Fe_2_O_3_ NPs (**A**) and biochar (**B**)

For biochar synthesis, the air-dried straw was ground into powder. The raw materials were pyrolyzed in a tube furnace under N_2_ atmosphere at 500 °C for 5 h. The obtained biochar was ground and passed through a 40-mesh screen prior to characterization. The scanning electron microscope (SEM; S-4800, Japan) images of biochar and α-Fe_2_O_3_ NPs are shown in Fig. 1. The XRD patterns of α-Fe_2_O_3_ NPs and biochar are shown in Additional file 1: Figure S1. Briefly, Crystallinity of the α-Fe_2_O_3_ NPs and biochar were tested by producing X-ray diffraction (XRD) patterns with an X-ray diffractometer (XRD; Bruker D8 Advance, Germany). The 2θ was within range of 10°–70°. Ni-filtered Cu Ka radiation was used as the X-ray source. Voltage and current were 40 kV and 40 mA, respectively. The hydrodynamic diameter and zeta potential of α-Fe_2_O_3_ NPs and the FTIR results of biochar were described in our previous study [24, 25].

## Greenhouse experimental design

A greenhouse experiment was designed to investigate individual and combined effects of Fe_2_O_3_ NPs and biochar on Cd treated muskmelon. Our previous results have demonstrated that muskmelon is a Cd-tolerant plant; thus, the selected exposure concentrations of Cd and the amendments, Fe_2_O_3_ NPs and biochar, were increased [25]. Details of each treatment are listed in Table 1. Muskmelon seeds were germinated in deionized water. Then, seedlings with uniform size were transplanted into pots containing matrix soil amended with different analytes to make the designated treatment. Muskmelon leaves at the same position in each seedling were sampled to analyze physiological and biochemical indicators. In addition, plant height, hypocotyl thickness, and leaf length and leaf width of muskmelon plants were measured at week 3, 5, 9 and 10. The size and weight of the muskmelon fruit was measured at harvest, and soil samples across all the treatments were also collected for Cd and Fe content analysis.

**Table 1:** Details of nine treatments in the pot experiment

Treatment	Exposure doses
Control (CK)	–
Cd	400 mg kg^−1^ Cd
FeNPs	400 mg kg^−1^ Cd + 50 mg kg^−1^ Fe
1%BC	400 mg kg^−1^ Cd + 1% biochar
3%BC	400 mg kg^−1^ Cd + 3% biochar
5%BC	400 mg kg^−1^ Cd + 5% biochar
1%BFNC	400 mg kg^−1^ Cd + 50 mg kg^−1^ Fe + 1% biochar
3%BFNC	400 mg kg^−1^ Cd + 50 mg kg^−1^ Fe + 3% biochar
5%BFNC	400 mg kg^−1^ Cd + 50 mg kg^−1^ Fe + 5% biochar

### Physiological measurements

Plant height, hypocotyl thickness, leaf length and leaf width were measured at week 3, 5, 9 and 10. At each testing point, the leaves at node 8, 12, 16 and 20 of each muskmelon were sampled for physiological and biochemical analysis.

At harvest, the fresh weight of fruits was measured using a FA1004C electronic analytical balance (Shanghai Yueping Scientific Instrument Co., Ltd, China). The vertical diameter, horizontal diameter and peel thickness of the fruits were also measured across all the treatment. The sugar content of muskmelon fruits was measured using a PAL-1 sugar detector (ATAG0).

For protein and MDA content measurements, approximately 0.6 g leaves were ground in phosphate buffer (PBS, 0.05 mM, pH 7.8), and then the mixtures were centrifuged at 4000 rpm, 4 ºC for 10 min. The supernatant was diluted to 10 mL with PBS (pH 7.8). The soluble protein content was measured using Coomassie Brilliant Blue G250 at 595 nm [24]. The MDA content was determined by thiobarbituric acid at 450, 532 and 600 nm using a UV-752 N spectrophotometer (Shanghai Precision Scientific instrument Co Ltd, China) [25].

### Pigment measurement

Chlorophyll content was measured using a SPAD-502 Plus chlorophyll meter (Konica Minolta). The leaves at three identical positions of each seedling were selected to measure the pigment content. For anthocyanin content measurements, approximately 0.1 g leaf tissues were ground in 1% (*v:v*) HCl (35%): ethanol (95%) solution. After the solution sits for 30 min, the mixtures were centrifuged at 4000 rpm for 10 min. The supernatant was diluted to 10 mL with 1% (*v:v*) HCl (35%): ethanol (95%) solution. The anthocyanin content was measured at 530 and 657 nm [29].

### Antioxidant enzyme activity

The antioxidant enzyme extracts were the same as used in the MDA content measurement. Superoxide dismutase (SOD) activity was determined using riboflavin and nitrogen blue tetrazole at 560 nm. For the peroxidase (POD) activity, H_2_O_2_ was used to initiate the reaction between POD and guaiacol, the absorbance of generated brown substance was measured at 470 nm every 30 s over 4.5 min, and the slope of the increased absorbance readings was used to calculate the POD activity. The catalase (CAT) activity was measured by calculating the H_2_O_2_ decomposition at 240 nm every 10 s over 1.5 min, the slope of the decreased absorbance readings was used to calculate the CAT activity [30]. For the Ascorbic acid peroxidase (APX) activity, APX was used to catalyze the reaction between H_2_O_2_ and ascorbate (ASA), the absorbance of remaining ASA was measured at 290 nm every 10 s over 1.5 min, and the slope of the decreased absorbance readings was used to calculate the APX activity [31].

### Cd and Fe content measurement

Plant tissues and soil samples were oven-dried and ground into fine powder. A weight of 0.15 mg sample was added into a tube containing 3 mL nitric acid and then heated at 100℃ for 1 h. After cooling to ambient temperature, 0.5 mL H_2_O_2_ were added for another half an hour at 100℃ to complete the digestion. The digesta were filtered and diluted with deionized water. For the available Cd in the soil, the soil samples were extracted with diethylenetriamine pentaacetic acid (DTPA), and then acidified with nitric acid for analysis [32]. The Cd and Fe concentration in digesta were determined by Avanta M atomic absorption spectrophotometer (GBC, Australia) [33].

### SEM–EDS analysis of Cd and Fe element in muskmelon tissues

Scanning electron microscope-energy dispersive spectrometer (SEM–EDS) (SEM, S-4800, Japan; EDS, GENESIS, USA) was used to further verify the presence of Fe and Cd in leaves and fruits. The freeze-dried leaves and fruit tissues were ground into fine powder and then mounted onto a sample stage for SEM–EDS analysis.

### RNA-Seq analysis of leaves upon exposure to Cd and two amendments

The 5-week old Cd treated muskmelon leaves were selected for transcriptomic analysis as affected by biochar and α-Fe_2_O_3_ NPs. Three biological replicates in the control and each treatment were applied. Thus, total number of 27 samples from 9 treatments were analyzed for transcriptome sequencing at Wuhan Genoseq Technology Co., Ltd., China. The total RNA in the sample was extracted using RNAprep Pure Plant kit (Bio TeKe, Beijing, China). The purity and concentration of RNA samples were determined using NanoDrop 2000 spectrophotometer (Thermo Scientific, Wilmington, DE, USA). Then the same amount of RNA from each sample was combined to construct a cDNA library. After digestion, the Hiseq 4000 (Illumina, San Diego, CA, USA) platform was used to sequence the first strand of cDNA. After data quality control, the clean data were aligned to the reference genome sequences of the muskmelon genome DHL92-v3.6.1. The differentially expressed genes (DEG) were identified in the R software through deseq2, with an FDR (false discovery rate) 0.05 and |log2 (fold change)|> 1 between two samples. The Gene Ontology (GO) and Kyoto Encyclopedia of Genes and Genomes (KEGG) enrichment analysis of differentially expressed genes were carried out by phyper in the R software, and the 20 most significant enrichment pathways were selected to draw the KEGG enrichment scatter plot.

### Statistical analysis

For each assay, the value is expressed as mean ± standard deviation (SD). A one-way analysis of variance followed by Duncan's multiple comparisons was used to test the significance at p < 0.05 (IBM SPSS version 22). Principal component analysis is a collection of the results of all the previous detection indicators, from which two main components are extracted to analyze the difference across all the treatments, and its visualization was completed by R 4.0.0. According to the results of transcriptome sequencing, the differentially expressed genes (DEG) were screened out according to the screening conditions of false discovery rate (FDR) < 0.05 and log_2_(fold change) ≧ 1, the DEG were enriched according to the different pathway, and then used R 4.0.0 to complete the visual drawing.

## Results and discussion

### Cd accumulation in plant tissues and soil

**Fig. 2:**
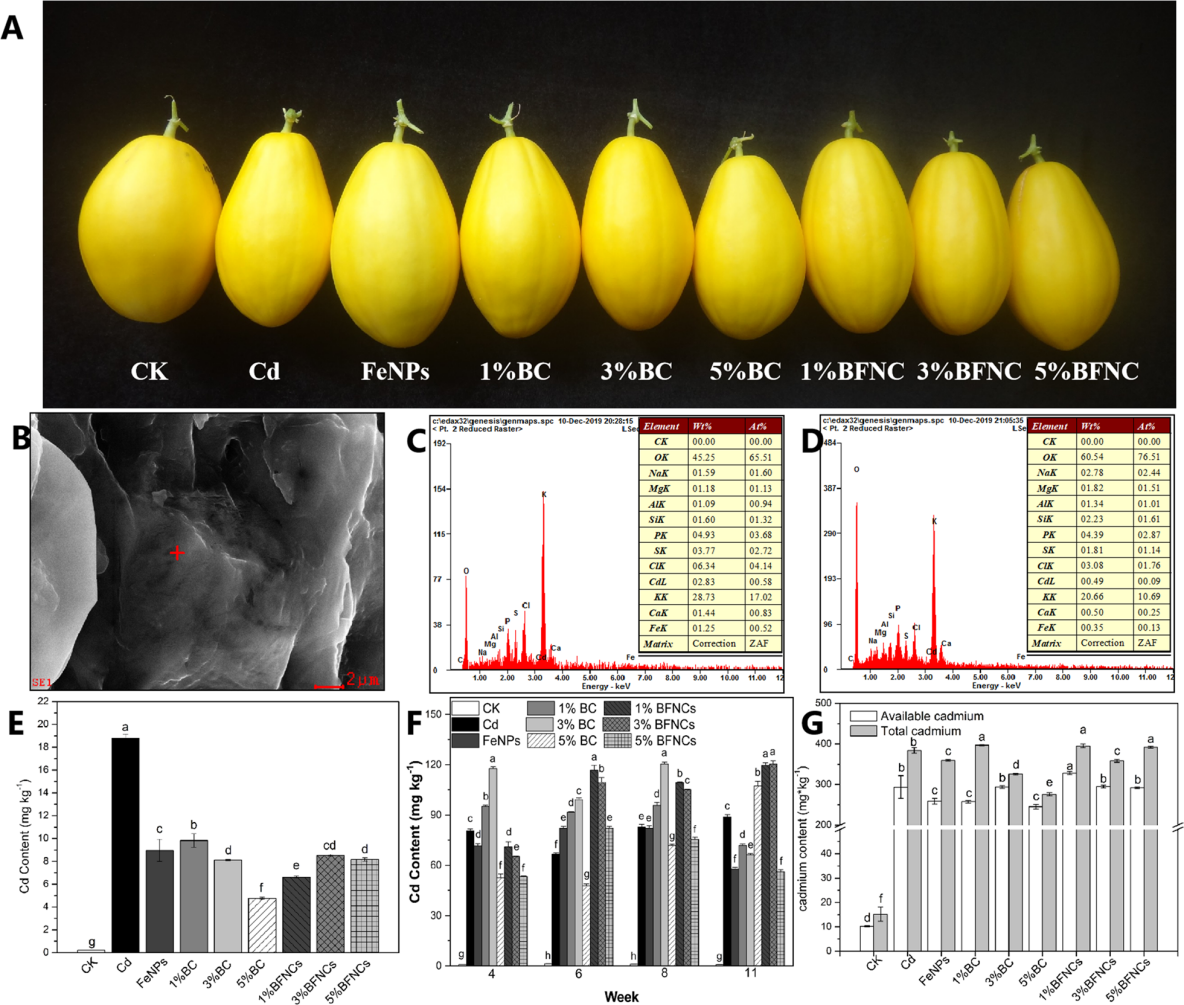
Cd accumulation in soil and muskmelon tissues. Phenotypic images of muskmelon fruits across all the treatments (**A**), SEM scanning images of muskmelon fruits in Cd-alone treatment (**B**), EDS analysis of Cd in muskmelon leaves in Cd-alone treatment (**C**) and FeNPs treatment (**D**), Cd content in muskmelon fruits (**E**), leaves (**F**) and soil (**G**) across all the treatments. Each bar represents the mean ± SD of three replicates. Different lowercase letters indicate significant differences at p < 0.05

The Cd content in muskmelon fruits of all treatments except control was higher than the national standard concentration of Cd (0.05 mg/kg) because of the extremely high Cd dose used in the present study. The Cd content (Fig. 2) decreased following the order: soil > leaves > fruits. In the Cd-alone treatment, the fruit Cd content was 4.7% of the soil Cd content. In the FeNPs, BC and BFNC treatments, the fruit Cd content was only 1.19–2.46% of the soil Cd content, suggesting that the single or co-treatments could significantly lower the Cd accumulation in the edible portion.

In addition, the biochar (1% or 5%) or α-Fe_2_O_3_ NPs treatment significantly reduced soil available Cd content, thereby reducing the Cd uptake by plants. The amendment of biochar potentially altered the hydrolysis balance of Cd and stabilized the soil Cd via complexation and precipitation [34]. Similarly, Fan et al. found that the main mechanism of the Cd adsorption process by biochar involved ion exchange, surface complexation, electrostatic interaction, surface co-precipitation, and other mechanisms [35]. After adding the nano iron oxide, Cd could bind directly to hydroxyl surface groups of Fe oxides [36], In addition, the iron plaque on root surfaces could act as a barrier to prevent contaminants from entering plant bodies [37, 38]. Thus, the effect of α-Fe_2_O_3_ NPs was well, too. However, the co-treatments with biochar and α-Fe_2_O_3_ NPs did not show an additive effect on the reduction of the soil available Cd content. Wu et al. (2019) reported that a portion of the micropores in the straw biochar was filled with γ-Al_2_O_3_ particles [39]. It is noted that the pore size of biochar (Fig. 1) was in micrometer, which was larger than the size of α-Fe_2_O_3_ NPs. Thus, it is reasonable that these large pores could be filled with nano-sized particles, and we also confirmed this view by SEM (Additional file 1: Figure S2). The presence of α-Fe_2_O_3_ NPs lowered the efficiency of biochar on the adsorption of the soil available Cd. In the BFNC treatments, the addition of 5% biochar reduced the Cd content in leaves to the similar level of the BC treatments because that the excess biochar might offset the negative effects caused by α-Fe_2_O_3_ NP adsorption by the biochar.

A decreasing pattern of the Cd content from leaves to fruits was evident (Fig. 2), indicating that more Cd was stored in the leaves. It is well-known that vacuole is the largest cell organelle and can store toxins to lower their toxicity to plants [40]. Previous study demonstrated that Cd complexed with chelatin was stored in the vacuoles, resulting the high concentration of Cd in the vacuole than in other cell organelles [41]. Energy-dispersive spectrum (EDS) analysis (Additional file 1: Figure S3 and Fig. 2C, D) further confirmed the Cd accumulation in the muskmelon fruits.

### Fe accumulation in muskmelon plants and soil

The Cd addition inhibited the Fe accumulation in muskmelon fruits (Additional file 1: Figure S4). However, across all the treatments with single or co-treatments, the Fe level in the Cd treated fruits was significantly increased when comparing to the Cd-alone treatment. It could be ascribed as that the competition between Cd and Fe was determined by the regulation of metal-related transporters [42]. Cd-induced mineral nutrient displacement could further affect plant metabolisms [43]. All the single or co-treatments significantly increased the Fe content in the fruits as compared to the Cd-alone treatment.

### Muskmelon growth and fruit traits

**Fig. 3:**
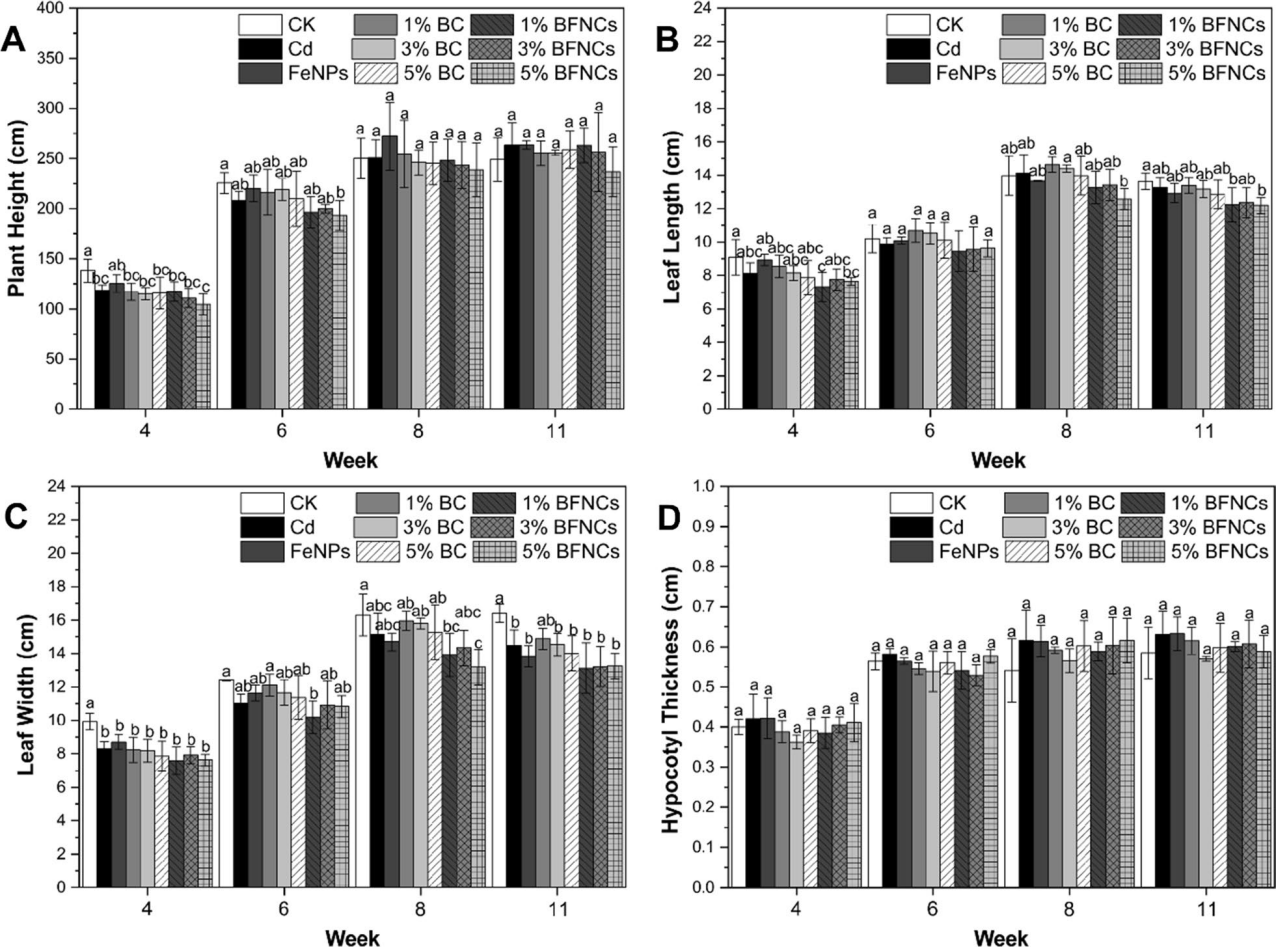
The growth record of muskmelon plants in the whole life cycle. Dynamic changes of plant height (**A**), leaf length (**B**), leaf width (**C**) and hypocotyl thickness (**D**) of muskmelon plants. The data show the mean ± SD of three replicates. Different lowercase letters indicate significant differences at p < 0.05

During the whole growth cycle, the plant height in all the treatments was significantly lower than the control at the early stage. With the growth of muskmelon plants, the plant height in all single or co-treatments was recovered to a certain extent compared to the control (Fig. 3A). The leaf width in all the treatments was significantly lower than that of the control during the whole growth period (Fig. 3C). Similarly, other physiological parameters, including fresh weight, sugar content, transverse diameter, and peel thickness, were all lower as compared to the control (Table 2), suggesting that the Cd-alone and the co-exposure with biochar and α-Fe_2_O_3_ NPs resulted in the certain levels of abiotic stresses to muskmelon plants. The size of the fruits across all the treatments was also consistent with the physiological results (Fig. 2A). In addition, no significant difference in the other growth indicators of all co-treatments as compared to the Cd-alone treatment, except for the fruit transverse diameter in the BFNC treatments, which was not only significantly smaller than that of the control, but also notably smaller than the ones in the Cd-alone treatment, indicating that the co-treatments with both biochar and α-Fe_2_O_3_ NPs might have an adverse effect on the Cd treated muskmelon to some extent.

**Table 2:** Size and quality of muskmelon fruits across all the treatments

Treatment	Fresh weight (kg/fruit)	Sugar content (%)	Vertical diameter (cm)	Transverse diameter (cm)	Peel thickness (cm)
Control	0.4656 a	14.6550 a	13.7683 a	8.8233 a	1.9900 a
Cd	0.3989 bc	13.5833 abc	13.3444 a	8.4806 b	1.8028 b
FeNPs	0.4044 bc	12.8017 bc	13.4100 a	8.5733 ab	1.7700 b
1% BC	0.4104 b	13.0611 bc	13.4139 a	8.5556 ab	1.8417 b
3% BC	0.4032 bc	13.0778 bc	13.5750 a	8.4722 b	1.7472 b
5% BC	0.3883 bc	12.8156 bc	13.4017 a	8.3439 bc	1.7461 b
1% BFNC	0.3548 c	13.4278 abc	12.7778 a	8.1083 c	1.7389 b
3% BFNC	0.3503 c	13.9233 ab	12.7233 a	8.1167 c	1.7300 b
5% BFNC	0.3530 c	12.2700 c	13.1100 a	8.1050 c	1.7717 b

### Physiological responses of muskmelon plants

#### Chlorophyll content

**Fig. 4:**
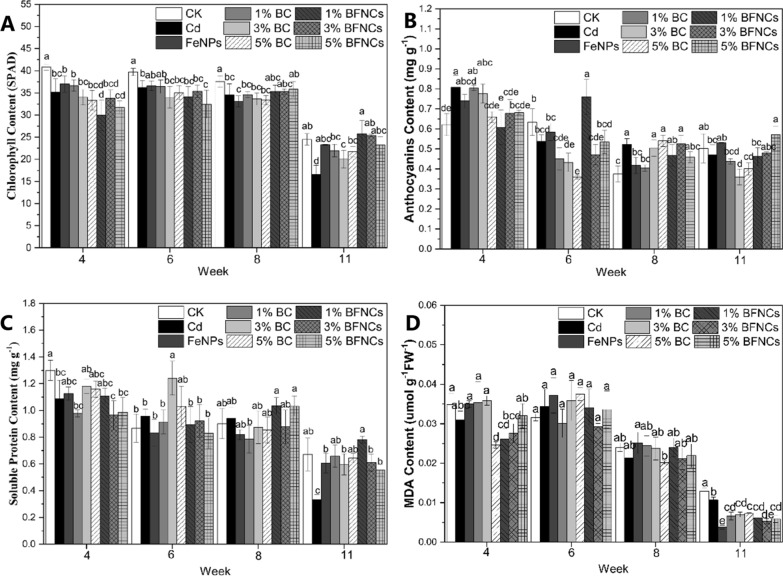
Physiological responses of muskmelon as affected by Cd, biochar and α-Fe_2_O_3_ NPs during the whole life cycle. Dynamic changes of chlorophyll (**A**), anthocyanin (**B**), soluble protein (**C**) and MDA content (**D**) in muskmelon plants. The data show the mean ± SD of three replicates. Different lowercase letters indicate significant differences at p < 0.05

During the whole life cycle, the chlorophyll content in the muskmelon leaves of the Cd alone treatment was significantly lower than that of the control (Fig. 4A). Consistently, R Mera et al. (2016) also demonstrated that the addition of Cd resulted in the chlorophyll reduction in the microalga Chlamydomonas moewusii [44]. Previous studies also demonstrated that Cd [2]^+^ ions can replace Mg^2+^ in chlorophyll (Chl) to form [Cd]-Chl [45] and subsequently compromised photosynthesis due to the Cd-induced reduction of the chlorophyll content, which was unstable [42]. The chlorosis of leaves in muskmelon plants treated with Cd alone was also evident (Additional file 1: Figure S5). The decrease in the chlorophyll content resulted in lowering the photosynthetic efficiency, and consequently inhibiting the muskmelon growth, which aligns with the results of physiological parameters throughout the whole life cycle. The chlorophyll content in the treatments with α-Fe_2_O_3_ NPs, 1%BC and 5%BC were no difference with the control at the eleventh week while the chlorophyll content in the Cd-alone treatment was still lower than these treatments, indicating that these three treatments had a positive effect on alleviating the decrease in chlorophyll content caused by cadmium stress.

#### Soluble protein content

Soluble protein is an important osmotic adjustment substance and nutrient substance in plants and contains various enzymes involved in metabolisms [46, 47]. It is often used as a phyisological indicator of plant resistance as the soluble protein synthesis is related to the resistance mechanisms of plants under abiotic stresses [33]. At the fourth and eleventh week, the soluble protein content in muskmelon leaves in the Cd-alone treatment was significantly lower by 16.5 and 50.5% as compared to the control. At the fourth week, the soluble protein content in the BC treatments and FeNPs treatment recovered to a comparable level with the control. At the eleventh week, the soluble protein content of muskmelon leaves in the single or co-treatments were restored to the control level.

### Antioxidant systems

Abiotic stresses in plants can generate excessive amounts of reactive oxygen species (ROS) accumulation, which can result in oxidative stresses, including lipid peroxidation, membrane leakage, enzyme inactivation, and DNA damage [48]. CAT, POD, SOD, APX and anthocyanins are primary antioxidant substances that are in charge of breaking down ROS [49, 50].

Excessive amounts of ROS in cells can lead to membrane lipid peroxidation and membrane leakage, resulting in a significant increase in MDA content [51]. However, in the present experiment, high dose of Cd did not significantly induce the MDA content (Fig. 4D).

**Fig. 5:**
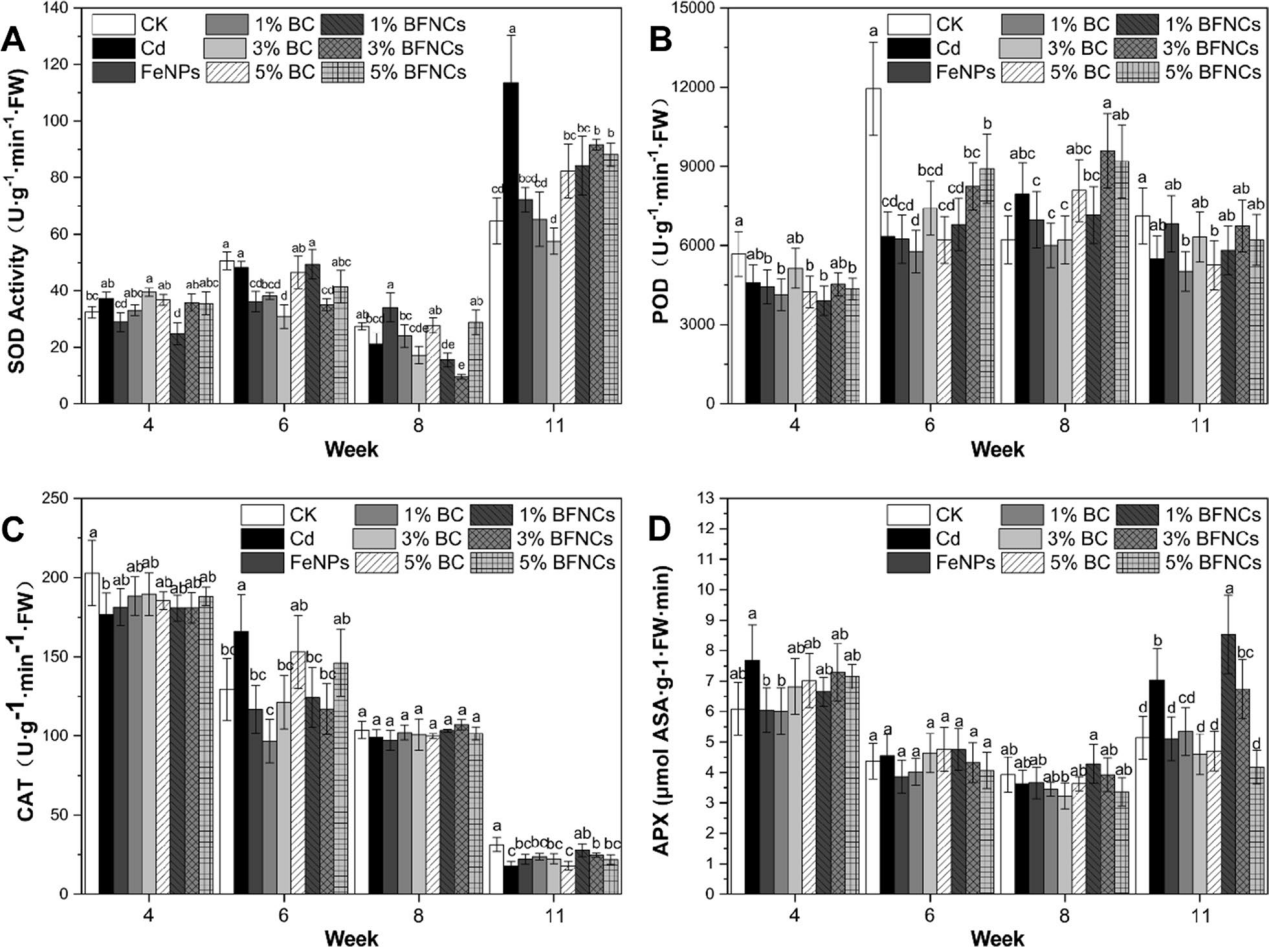
Antioxidant enzyme activities of muskmelon plants as affected by Cd, biochar and α-Fe_2_O_3_ NPs during the whole life cycle. Dynamic changes of SOD (**A**), POD (**B**), CAT (**C**) and APX activities (**D**) in muskmelon plants. The data show the mean ± SD of three replicates. Different lowercase letters indicate significant differences at p < 0.05

At the 11th week, the SOD and APX activities in leaves treated with Cd-alone were significantly increased as compared to the control; while the POD activity at the sixth week and the CAT activity at the 4th and 11th weeks were significantly decreased (Fig. 5). Previous studies reported that a dose-dependent pattern of the antioxidant enzyme activity was evident; the lower the Cd doses were, the higher the activities of antioxidant enzymes were found in plants [52]. In a relatively high Cd dose scenario, the various responses of the activities of four antioxidant enzymes indicate inconsistent sensibility to the Cd-induced phytotoxicity [53]. The reduction of antioxidant enzyme activity will inevitably lead to an increase in intracellular reactive oxygen species, which can also be reflected by the increase in anthocyanin content (Fig. 4B).

At the fourth and eighth weeks, the anthocyanin content (Fig. 4B) in Cd-alone treated leaves was significantly increased by 30.03 and 39.36% as compared to the control, respectively. In the FeNPs treatment, the anthocyanin content was decreased to the control level. Similarly, 5% biochar treatment at week 4 and 1% biochar treatment at week 8 also produced the same effect. The SOD and APX (Fig. 5A and D) activities in the Cd-alone treated leaves increased significantly, but were only found at the eleventh week; while their activities in FeNPs and BC treatments were recovered to the control level. At the eleventh week, the SOD activity in leaves in the BFNC treatments was significantly lower than that in the Cd-alone treatment, but still significantly higher than the control. The APX activity in FeNPs and BC treatments were recovered to the control level at the eleventh week.

At the sixth week, the activity of POD in the Cd-alone treatment was significantly reduced by 50% compared to the control. With the 5% BFNC treatment as an exception, whose POD activity was significantly higher than the Cd-alone treatment (Fig. 5B), there was no significant difference in the other remediation treatment with Cd alone treatment. The POD activity in the Cd-alone treatment also decreased insignificantly compared to the control at the fourth and eleventh weeks. Similarly, at the fourth and eleventh weeks, the CAT activity in the Cd-alone treatment was significantly lower than that of the control. At the eleventh week, in the co-treatments with 1% and 3% biochar, the CAT activities were significantly elevated when comparing to the Cd-alone treatment.

In the experiment, the responses of the four antioxidant enzymes to Cd stress were not consistent, which may be due to differences in their sensitivity to Cd [54]. In addition, most of the oxidative stress caused by Cd stress occur in the seedling and mature stages of muskmelon, which may be attributed to the plant's own regulatory effects [55].

### Principal component analysis

Principal component analysis (PCA) is a method of dimensionality reduction analysis, referring to extracting one or two main components from a large number of indicators [56]. Through principal component analysis, we can better understand the differences between samples and the correlation between indicators.

In the experiment, we extracted two main components, covering more than 50% of all the changes across all the treatments and parameters. In the fourth and sixth weeks, Cd content was negatively correlated with leaf length, leaf width, plant height and hypocotyl thickness, indicating that Cd stress inhibited the growth of muskmelon plants, which was consistent with our previous results. Cd content and chlorophyll content were negatively correlated throughout the growth cycle, indicating that Cd inhibited the synthesis of chlorophyll in muskmelon leaves. Regarding the antioxidant systems, in general, Cd stress was positively correlated with the activities of SOD and APX during most of the growth period of muskmelon plants, but negatively correlated with the activities of CAT and POD.

**Fig. 6:**
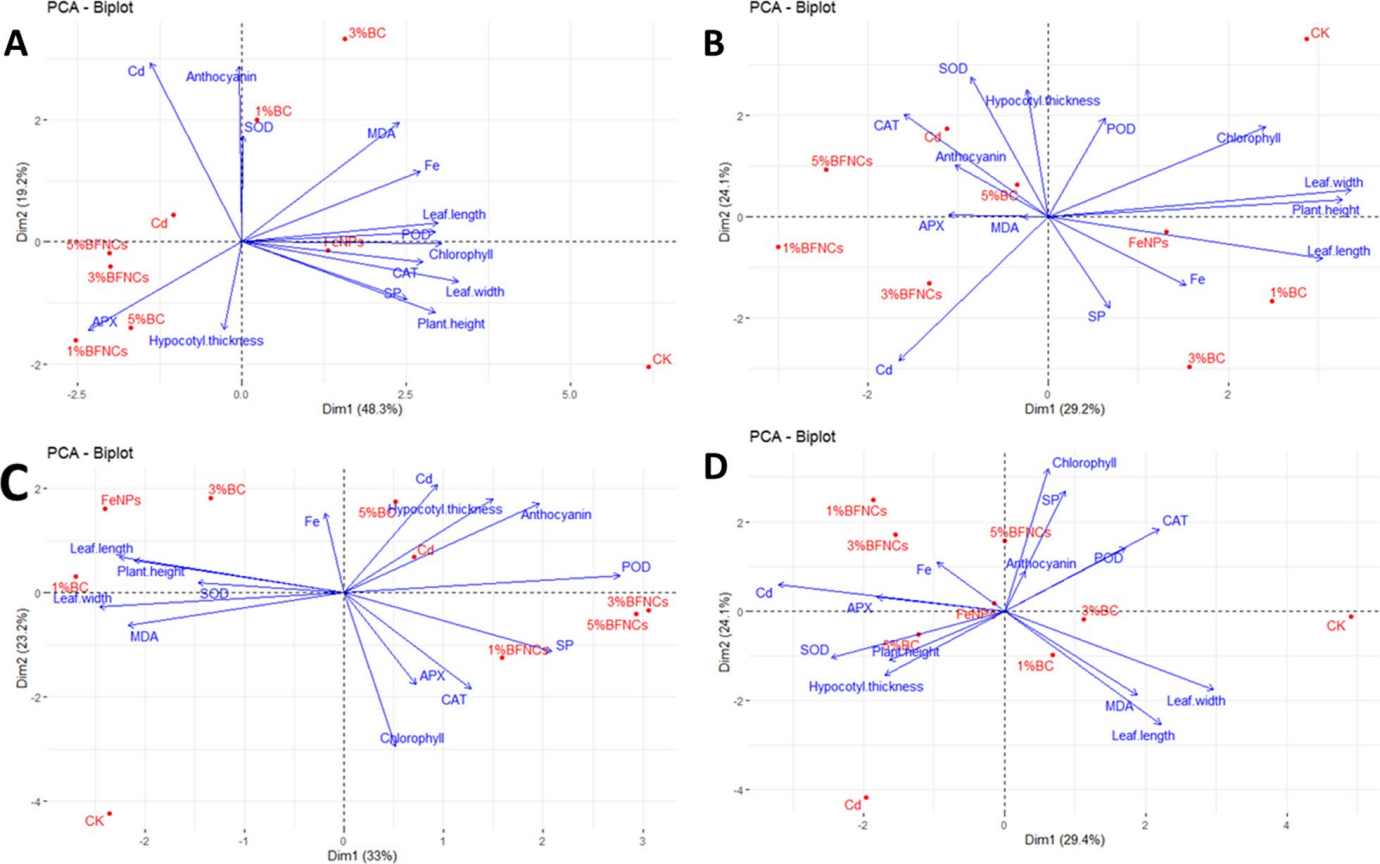
Principal component analysis of all physiological parameters in muskmelon plants. Principal component analysis was conducted on the physiological indicators of muskmelon plants in the fourth (**A**), sixth (**B**), eighth (**C**), and eleventh (**D**) weeks. The horizontal axis is the extracted first and second principal components, respectively

From the distribution of the treatment groups in the Fig. 6, Cd-alone treatment had a significant impact on the growth of plants, and all Cd-containing treatments had a greater impact on the growth of muskmelon. From the week 4 to the week 11, the groups with single or co-treatments gradually moved away from the Cd treatment group, indicating that with the growth of muskmelon plants, both single or co-treatments alleviated the Cd stress to a certain extent. In most of the time (Fig. 5A, B and C), single and co-treatments were in different quadrants and were far apart, indicating that in contrast, single treatments had a better repair effect on Cd stress, which was consistent with our previous analysis.

### Transcriptome analysis

**Fig. 7:**
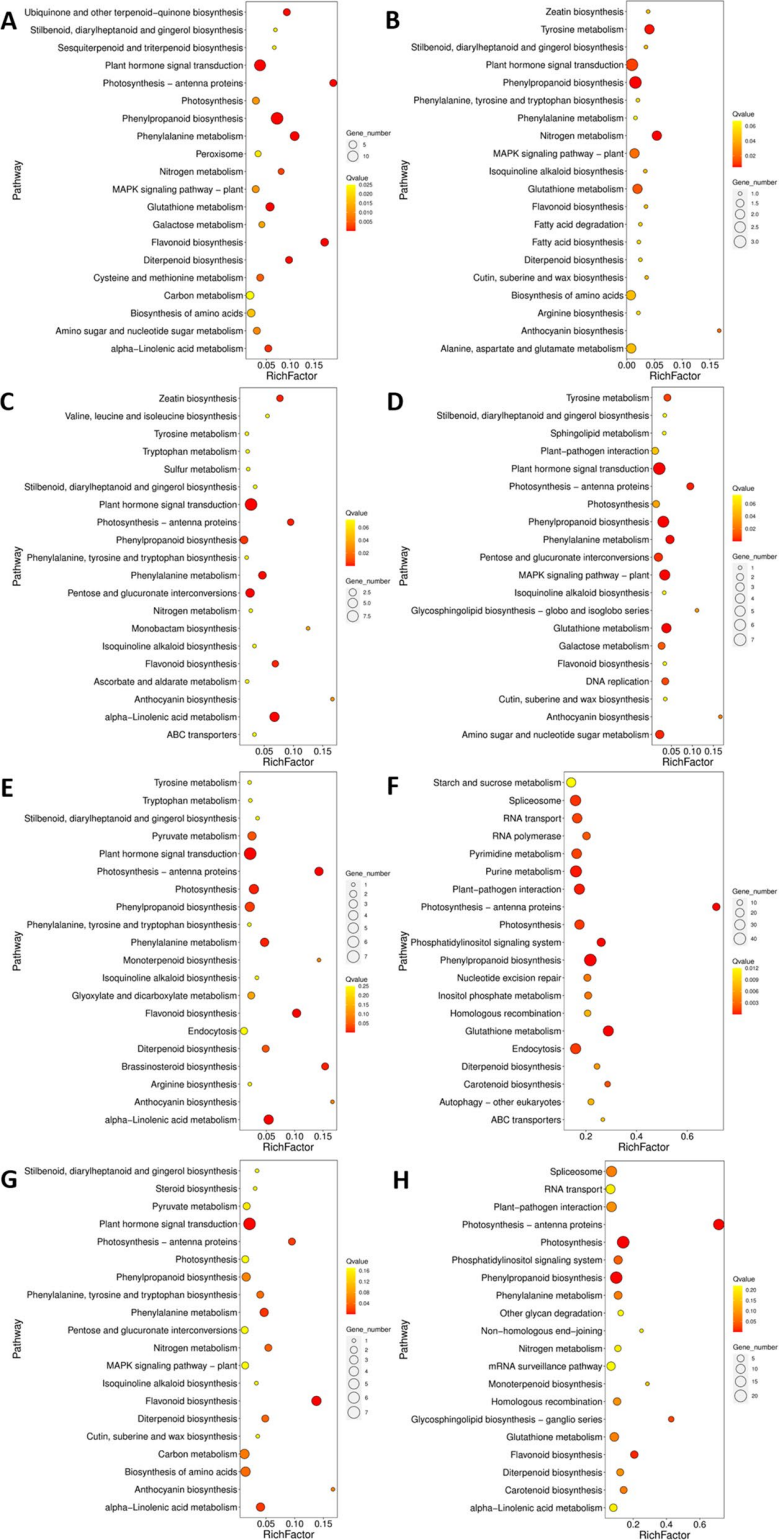
Enrichment of differentially expressed genes in muskmelon plants. The enrichment results of different genes in Cd (**A**), FeNPs (**B**), 1%BC (**C**), 3%BC (**D**), 5%BC (**E**), 1%BFNC (**F**), 3%BFNC (**G**) and 5%BFNC (**H**) treated muskmelon plants as compared to the control

RNA-Seq technique was used to reveal the transcriptomic response of muskmelon leaves as a function of Cd, biochar and α-Fe_2_O_3_. In comparison with the control, 394 genes were significantly different in the Cd-alone treatment. Among them, 301 genes were up-regulated by more than twofold. The results of KEGG enrichment (Fig. 7) show that differentially expressed genes are related to multiple signal transduction pathways and biochemical metabolic processes. Among them, in the Cd-alone treatment, genes associated with photosynthesis were significantly down-regulated; while genes related to anthocyanin synthesis and glutathione metabolisms, both of which can scavenge ROS in plants [33], were significantly up-regulated. Genes related to sulfur metabolism were significantly down-regulated. Cysteine, an important hub of sulfur metabolism, is a substrate of glutathione and is involved in maintaining the level of antioxidant glutathione [57, 58]. In addition, genes related to the mitogen-activated protein kinase (MAPK) signaling pathway were significantly up-regulated. Previous study also demonstrated that the MAPK pathway could respond to the Cd-induced abiotic stress [59]. Heavy metal stresses can alter the patterns of gene regulation in plants [60]. MAPK is one of the main pathways that plants transduce extracellular stimuli and other signals to cause intracellular responses [61]. Given the evidence on the significant up-regulation of the gluthation metabolism associated genes, it is reasonable to speculate that exposure to Cd induces strong oxidative stress in the muskmelon at the early stage. Through MAPK or other signal transduction pathways, the expression of photosynthesis-related genes was down-regulated, and the synthesis and metabolism of antioxidant-related substances were triggred/activated to break down ROS in plants. However, the expression of genes related to sulfur metabolism was significantly down-regulated, and thus, the gluthatione biosynthesis as the down-stream metabolism pathways of sulfur assimilation might be affected as well.

In the remediation treatments, up-regulation of genes related to anthocyanin synthesis was found as compared to the control, indicating that even if biochar or Fe is added to the Cd pollution, thereis still a certain amount of stress on the muskmelon plants compared with the control. In the 1%BC treatment (Fig. 7C), the ABC transporter family related genes were found to be significantly up-regulated as compared to the control. *AtPDR8* (member of the pleiotropic drug resistance) in the ABC transporter family can act as an efflux pump for Cd^2+^ ions or Cd conjugates in *Arabidopsis* [62], implying that these two remediation treatments might indirectly alter the Cd distribution by effluxing Cd out of the plant body and subsequently alleviate the Cd-induced phytotoxicity. Similar KEGG enrichments in anthocyanin biosynthesis, glutathione metabolism and MAPK signal transduction pathways were also found in the treatments with α-Fe_2_O_3_ NPs and 3%BC. Therefore, it can be speculated that after the addition of the remediation agent, in addition to the aforementioned regulation of the muskmelon plant itself, it may also activate metal ion transporters such as the ABC transporter family, thereby expelling Cd ions to reduce the toxicity of Cd to cells. The differential expression of a large number of genes appeared in the 1%BFNC and 5%BFNC treatments, which was significantly more than other treatments. Through enrichment analysis of differential genes, we found that there were significant differential expressions of genes related to ABC transporter, anthocyanin biosynthesis, glutathione metabolism and MAPK signal transduction pathways, which could alleviate the Cd stress. It should be noted that the 3% BFNC treatment has fewer differential genes as compared to the control, implying 50 mg/kg α-Fe_2_O_3_ NPs and 3% biochar was a relatively good composite ratio. But combined with the previous growth and physiological indicators, single treatments had a better repair effect than co-treatments on Cd stress.

## Conclusions

Our research found that Cd pollution reduced the absorption of iron elements by muskmelon (*Cucumis melo*) plants (Additional file 1: Fig. S4), and also caused the growth of muskmelon plants to be inhibited (Fig. 3) and the size and quality of fruits to be reduced (Table 2). In addition, the high concentration of Cd pollution will also lead to a series of physiological and biochemical changes in muskmelon plants, including a decrease in chlorophyll and soluble protein content, an increase in anthocyanin content, and a decrease in POD and CAT activity. The addition of α-Fe_2_O_3_ NPs or biochar alone significantly reduced the absorption of Cd by plants, but the effect of co-treatments was not as good as that of the single treatments. The results of gene differential expression analysis showed that the expression of Cd transport related proteins in the repair treatments did increase significantly, which confirmed that the repair treatments had a certain positive effect on alleviating the Cd poisoning of plants. In addition, all treatments could significantly reduce the Cd content in fruit, and single treatments could alleviate the Cd toxicity according to various indicators. It should be noted that the compound treatment in our research does not seem to produce better results, but 50 mg/kg α-Fe_2_O_3_ NPs and 3% biochar was a relatively good composite ratio. Our research shows that biochar and α-Fe_2_O_3_ NPs can significantly reduce the effective Cd content in the soil and the Cd content in the fruit, which provides some ideas for the safe cultivation of muskmelon crops in Cd -contaminated soil. Our research provides some considerations for agricultural production and environmental protection under Cd pollution.

## Supplementary Information

**Additional file 1**: Additional figures and tables.

## Data Availability

All data associated with this study are present in the paper or additional materials.

## References

[1] Ali A, Guo D, Jeyasundar P, Li Y, Zhang Z (2019). Application of wood biochar in polluted soils stabilized the toxic metals and enhanced wheat (Triticum aestivum) growth and soil enzymatic activity. Ecotoxicology and Environmental Safety.

[2] Wu T, Liu C, Kong B, Sun J, Gong Y, Liu K, Xie J, Pei A, Cui Y (2019). Amidoxime-functionalized macroporous carbon self-refreshed electrode materials for rapid and high-capacity removal of heavy metal from water. ACS Cent Sci.

[3] Pa ND, Liu C, Yu H, Li F (2019). A paddy field study of arsenic and cadmium pollution control by using iron-modified biochar and silica sol together. Environ Sci Pollut Res.

[4] Qayyum MF, Rehman RA, Liaqat S, Ikram M, Hussain Q (2019). Cadmium immobilization in the soil and accumulation by spinach ( Spinacia oleracea ) depend on biochar types under controlled and field conditions. Arab J Geoences.

[5] Ali S, Rizwan M, Noureen S, Anwar S, Ali B (2019). Combined use of biochar and zinc oxide nanoparticle foliar spray improved the plant growth and decreased the cadmium accumulation in rice (*Oryza sativa* L.) plant. Environm Sci Pollut Res.

[6] Khan MA, Islam MR, Panaullah GM, Duxbury JM, Loeppert J (2010). Accumulation of arsenic in soil and rice under wetland condition in Bangladesh. Plant Soil.

[7] Rizwan M, Ali S, Rehman M, Adrees M, Arshad M (2019). Alleviation of cadmium accumulation in maize (*Zea mays* L.) by foliar spray of zinc oxide nanoparticles and biochar to contaminated soil. Environ Pollut.

[8] Ogawa T, Kobayashi E, Okubo Y, Suwazono Y, Kido T, Nogawa K (2004). Relationship among prevalence of patients with Itai-itai disease, prevalence of abnormal urinary findings, and cadmium concentrations in rice of individual hamlets in the Jinzu River basin, Toyama prefecture of Japan. Int J Environ Health Res.

[9] Kamran M, Malik Z, Parveen A, Zong Y, Ali M (2019). Biochar alleviates Cd phytotoxicity by minimizing bioavailability and oxidative stress in pak choi (*Brassica chinensis* L.) cultivated in Cd-polluted soil. J Environ Manag.

[10] Liu X, Zeng Z, Tie B, Chen Q, Chen Z, Zheng R (2013). Effects of biochar and lime application on soluble Cd, Pb, As release and non-point loads of rice agroecosystem by in situ field experiment, Central Hunan Province Mining Area. Hamdard Medicus.

[11] Abdelhamid HN, Mathew AP (2021). Cellulose-zeolitic imidazolate frameworks (CelloZIFs) for multifunctional environmental remediation: Adsorption and catalytic degradation. Chem Eng J.

[12] Fijoł N, Abdelhamid HN, Pillai B (2021). 3D-printed monolithic biofifilters based on a polylactic acid (PLA)-hydroxyapatite (HAp) composite for heavy metal removal from an aqueous medium[J]. RSC Adv.

[13] Nzediegwu C, Prasher S, Elsayed E, Dhiman J, Mawof A, Patel R (2018). Effect of biochar on heavy metal accumulation in potatoes from wastewater irrigation. J Environ Manag.

[14] Jin HP, Choppala GK, Bolan NS, Chung JW, Chuasavathi T (2011). Biochar reduces the bioavailability and phytotoxicity of heavy metals. Plant Soil.

[15] Younis U, Malik SA, Rizwan M, Qayyum MF, Ok YS, Shah M, Rehman RA, Ahmad N (2016). Biochar enhances the cadmium tolerance in spinach (*Spinacia oleracea*) through modification of Cd uptake and physiological and biochemical attributes. Environ Sci Pollut Res.

[16] Zhang ZY, Meng J, Dang S, Chen WF (2014). Effect of biochar on relieving cadmium stress and reducing accumulation in *Super japonica* rice. J Integr Agric.

[17] Woldetsadik D, Drechsel P, Keraita B, Marschner B, Itanna F, Gebrekidan H (2016). Effects of biochar and alkaline amendments on cadmium immobilization, selected nutrient and cadmium concentrations of lettuce (*Lactuca sativa*) in two contrasting soils. Springerplus.

[18] Rajendran M, Shi L, Wu C, Li W, An W, Liu Z, Xue S (2019). Effect of sulfur and sulfur-iron modified biochar on cadmium availability and transfer in the soil–rice system. Chemosphere.

[19] Liu XM, Zhang FD, Feng ZB, Zhang SQ, Xu-Sheng HE, Wang RF, Wang YJ (2005). Effects of Nano-ferric oxide on the growth and nutrients absorption of peanut. Plant Nutr Fertil.

[20] Zhang R, Chen W. Graphene-non-noble metal hybrid nanomaterials as advanced electrocatalysts. 2014.

[21] Palmqvist N, Seisenbaeva GA, Svedlindh P, Kessler VG (2017). Maghemite nanoparticles acts as nanozymes, improving growth and abiotic stress tolerance in *Brassica napus*. Nanoscale Res Lett.

[22] Zhu D, Jian Z, Song J, Wang H, Zheng Y, Shen Y, Xie A (2013). Efficient one-pot synthesis of hierarchical flower-like α-Fe2O3 hollow spheres with excellent adsorption performance for water treatment. Appl Surf Sci.

[23] Maia L, Santos MS, Andrade TG, Hott R, Rodrigues JL (2018). Removal of mercury(II) from contaminated water by gold-functionalised Fe3O4 magnetic nanoparticles. Environ Technol.

[24] Li J, Wan F, Guo W, Huang J, Wang Y (2020). Influence of α- and γ-Fe2O3 Nanoparticles on Watermelon (*Citrullus lanatus*) Physiology and Fruit Quality. Water Air Soil Pollut.

[25] Zou Z, Wang Y, Huang J, Lei Z, Wang F, Dai Z, Yi L, Li J (2020). A study on the mixture repairing effect of biochar and nano iron oxide on toxicity of Cd toward muskmelon. Environ Pollut.

[26] Alidoust D, Isoda A (2014). Phytotoxicity assessment of γ-Fe2O3 nanoparticles on root elongation and growth of rice plant. Environ Earth Sci.

[27] Fang G, Grumet R (1993). Transformation in Muskmelon (*Cucumis Melo* L.). Plant Protoplasts Genetic Eng IV.

[28] Geng X, Li K, Wang F, Pan T, Diao Y, Lin Y (2014). Extraction, purification and pharmacological activity of melon pedicle active ingredients. Asian J Chem.

[29] Lopez-Martinez LX, Oliart-Ros RM, Valerio-Alfaro G, Lee CH, Parkin KL, Garcia HS (2009). Antioxidant activity, phenolic compounds and anthocyanins content of eighteen strains of Mexican maize. Food Sci Technol.

[30] Wang Y, Wang S, Xu M, Xiao L, Dai Z, Li J (2019). The impacts of γ-Fe_2_O_3_ and Fe_3_O_4_ nanoparticles on the physiology and fruit quality of muskmelon (*Cucumis melo*) plants. Environ Pollut.

[31] Ma D (2011). Studies on salt tolerance of transgenic sweetpotato which harbors two genes expressing CuZn superoxide dismutase and ascorbate peroxidase with the stress-inducible SWPA2 promoter. Plant Gene Trait.

[32] Haq AU, Bates TE, Soon YK (1980). Comparison of extractants for plant-available zinc, cadmium, nickel, and copper in contaminated soils. Soil Sci Soc Am J.

[33] Xiao L, Guo H, Wang S, Li J, Wang Y, Xing B (2019). Carbon dots alleviate the toxicity of cadmium ions (Cd2+) toward wheat seedlings. Environ Nano.

[34] Ding W, Zhu Q, Zeng X, Dan WU (2011). Biochars from different pyrolytic temperature amending lead and cadmium contaminated soil. Sci Technol Rev.

[35] Fan S, Li H, Wang Y (2018). Cadmium removal from aqueous solution by biochar obtained by co-pyrolysis of sewage sludge with tea waste. Res Chem Intermed.

[36] Muehe EM, Obst M, Hitchcock AP (2013). Fate of Cd during microbial Fe(III) mineral reduction by a novel and Cd-tolerant Geobacter species. Environ Sci Technol.

[37] Colmer TD (2010). Long-distance transport of gases in plants: a perspective on internal aeration and radial oxygen loss from roots. Plant, Cell Environ.

[38] Chen RF, Shen RF, Gu P, Dong XY, Du CW, Ma JF (2006). Response of rice (*Oryza sativa*) with root surface iron plaque under aluminium stress. Ann Bot.

[39] Wu P, Cui P, Marcelo E (2019). Interactive effects of rice straw biochar and γ-Al2O3 on immobilization of Zn. J Hazard Mater.

[40] Krotz RM, Evangelou BP, Wagner GJ (1989). Relationships between cadmium, zinc, Cd-peptide, and organic acid in tobacco suspension cells. Plant Physiol.

[41] Salt DE, Rauser WE (1995). MgATP-dependent transport of phytochelatins across the tonoplast of oat roots. Plant Physiol.

[42] Andresen E, Küpper H (2013). Cadmium toxicity in plants. Met Ions Life Sci.

[43] Nazar R, Iqbal N, Masood A, Khan M, Khan NA (2012). Cadmium toxicity in plants and role of mineral nutrients in its alleviation. Am J Plant Sci.

[44] Mera R, Torres E, Abalde J (2016). Influence of sulphate on the reduction of cadmium toxicity in the microalga *Chlamydomonas moewusii*. Ecotoxicol Environ Saf.

[45] Hendrik K, Frithjof K, Martin S (1996). Environmental relevance of heavy metal-substituted chlorophylls using the example of water plants. J Exp Bot.

[46] Wang K, Guo JJ, Wang DQ, Liu S, Ni PP (2015). Responses of roots and needles of *Pinus sylvestris* var. mongolica and *Pinus tabuliformis* to spring drought stress. Chin J Ecol.

[47] Zhao MZ, Liu ZL, Chen W, Cai SY (2014). Responses of *Trifolium repens* cv. rivendel seeds to cadmium stress in terms of electrolyte leakage and soluble protein content changes. Adv Mater Res.

[48] Halliwell B, Gutteridge J (1985). Free radicals in biology and medicine. Biochem Soc Trans.

[49] Anjum SA, Tanveer M, Hussain S, Shahzad B, Ashraf U, Fahad S, Hassan W, Jan S, Khan I, Saleem MF (2016). Osmoregulation and antioxidant production in maize under combined cadmium and arsenic stress. Environ Sci Pollut Res.

[50] Xu Z, Rothstein SJ (2018). ROS-Induced anthocyanin production provides feedback protection by scavenging ROS and maintaining photosynthetic capacity in Arabidopsis. Plant Signal Behav.

[51] García A (2016). Vermicompost humic acids modulate the accumulation and metabolism of ROS in rice plants. J Plant Physiol.

[52] Sandalio LM, Dalurzo HC, Gómez M (2001). Cadmium-induced changes in the growth and oxidative metabolism of pea plants. J Exp Bot.

[53] Xue H, Zhang H, Chang H, Jiao Y, Li H (2015). Effect of cadmium on antioxidase system in process of maize germination. Asian J Chem.

[54] Batool M, Abdullah S, Abbas K (2014). Antioxidant enzymes activity during acute toxicity of chromium and cadmium to Channa marulius and Wallago attu. Pak J Agric Sci.

[55] Wang Y, Hu J, Dai Z, Li J, Huang J (2016). In vitro assessment of physiological changes of watermelon (*Citrullus lanatus*) upon iron oxide nanoparticles exposure. Plant Physiol Biochem.

[56] Amini A, Wainwright M (2008). High-dimensional analysis of semidefinite relaxations for sparse principal components. Ann Statist.

[57] Yanmei C, Yuanqing C, Yaying L, Qingqi L, Jun B (2016). Survival strategies of the plant-associated bacterium *Enterobacter* sp. strain EG16 under cadmium stress. Appl Environ Microbiol.

[58] Capaldi FR, Gratao PL, Reis AR, Lima LW, Azevedo RA (2015). Sulfur metabolism and stress defense responses in plants. Trop Plant Biol.

[59] Jonak C, Nakagami H, Hirt H (2004). Heavy metal stress. Activation of distinct mitogen-activated protein kinase pathways by copper and cadmium. Plant Physiol.

[60] Lin CW, Chang HB, Huang HJ (2005). Zinc induces mitogen-activated protein kinase activation mediated by reactive oxygen species in rice roots. Plant Physiol Biochem.

[61] Zhao FY, Han MM, Zhang SY, Ren J, Hu F, Wang X (2014). MAPKs as a cross point in H_2_O_2_ and auxin signaling under combined cadmium and zinc stress in rice roots. Russ J Plant Physiol.

[62] Kim DY, Bovet L, Maeshima M, Martinoia E, Lee Y (2010). The ABC transporter AtPDR8 is a cadmium extrusion pump conferring heavy metal resistance. Plant J.

