# Supplementary material for: Physiological of biochar and α-Fe2O3 nanoparticles as amendments of Cd accumulation and toxicity toward muskmelon grown in pots
Source: J Nanobiotechnology. 2021 Dec 20;19:442. doi: 10.1186/s12951-021-01187-7 (PMC8690976; doi:10.1186/s12951-021-01187-7)
Supplement: Supplementary file 1 — Additional file 1: Additional figures and tables. [file 12951_2021_1187_MOESM1_ESM.docx]

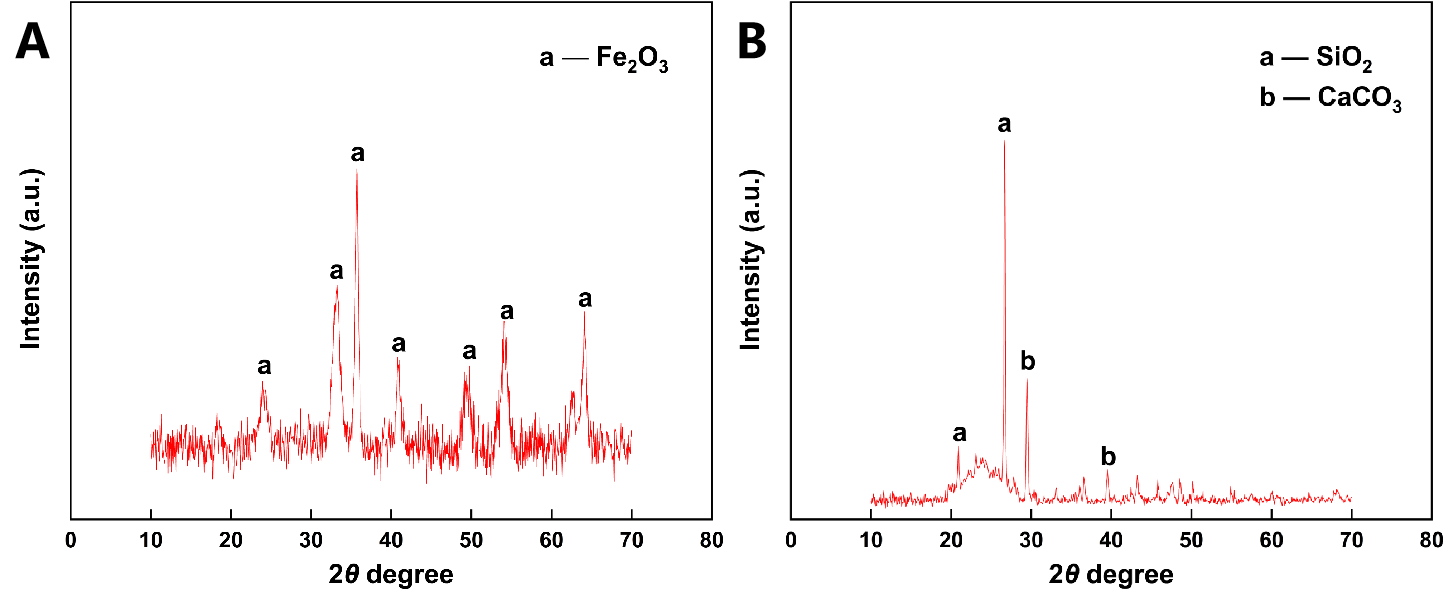


**Figure S1.** XRD patterns of α-Fe_2_O_3_ NPs (A) and biochar (B).

**
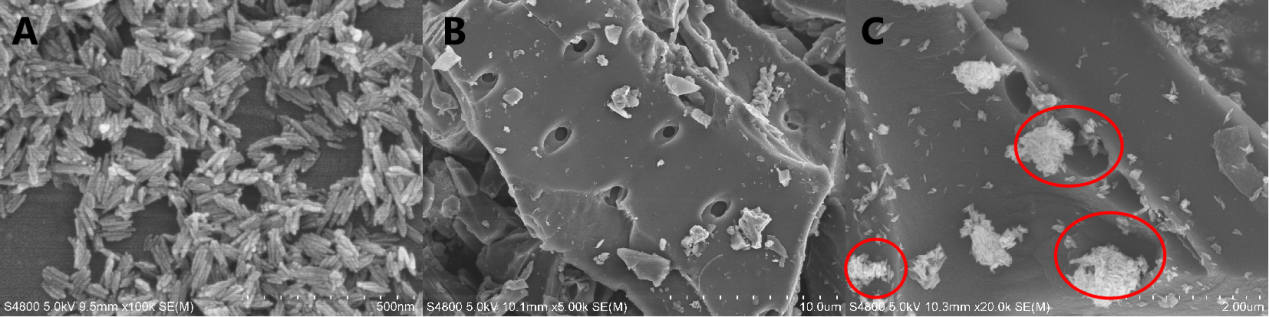
**

**Figure S2.** SEM images of α-Fe_2_O_3_ NPs (A), biochar (B) and combination of α-Fe_2_O_3_ NPs and biochar (C).

As can be seen from figure S2C, when α-Fe_2_O_3_ NPs and biochar are put together, these large pores of biochar could be filled with α-Fe_2_O_3_ NPs.

**
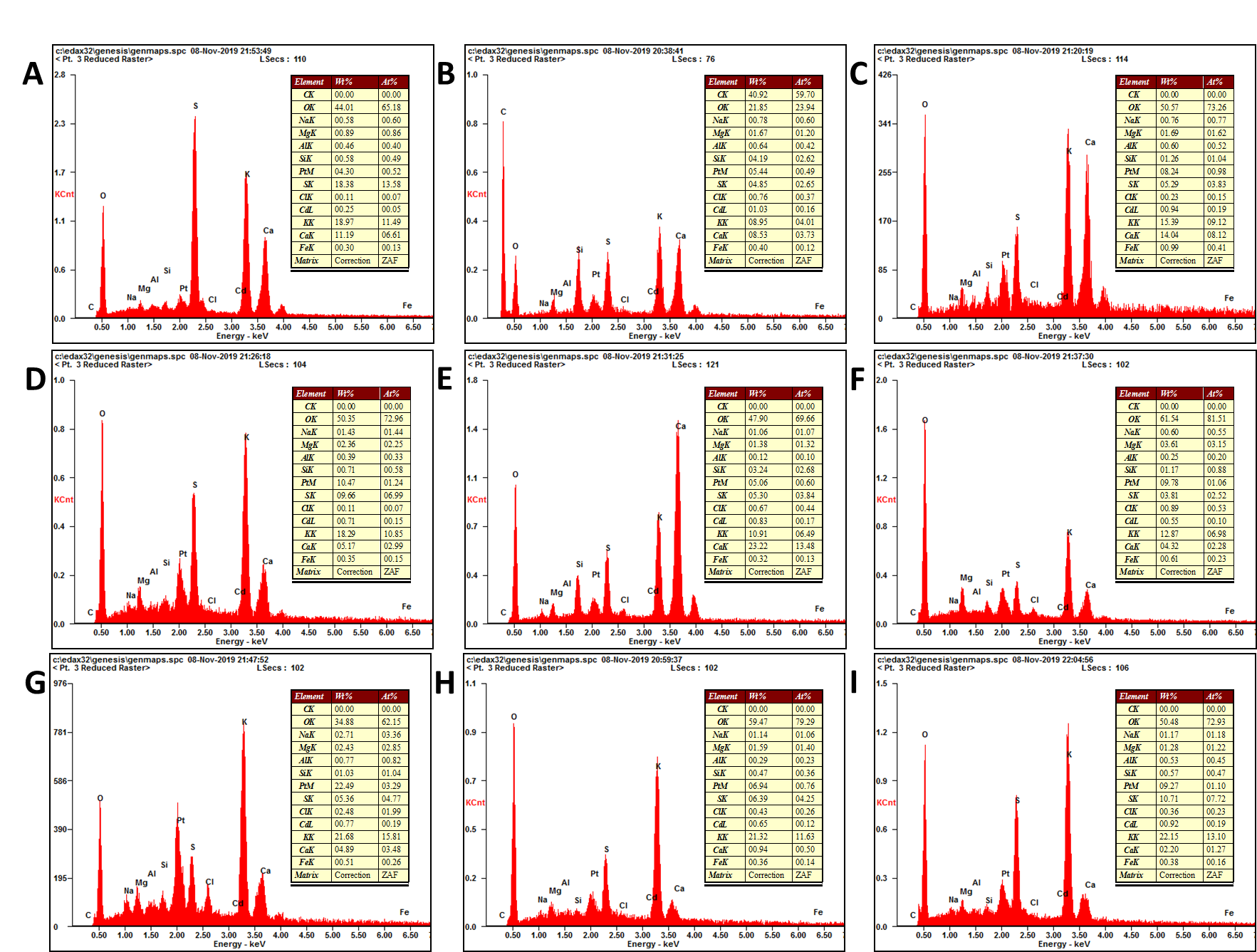
**

**Figure S3.** EDS analysis of melon leaves of each treatment group. A-I corresponds to nine experimental groups of CK, Cd, FeNPs, 1%BC, 3%BC, 5%BC, 1%BFNCs, 3%BFNCs and 5%BFNCs.

**
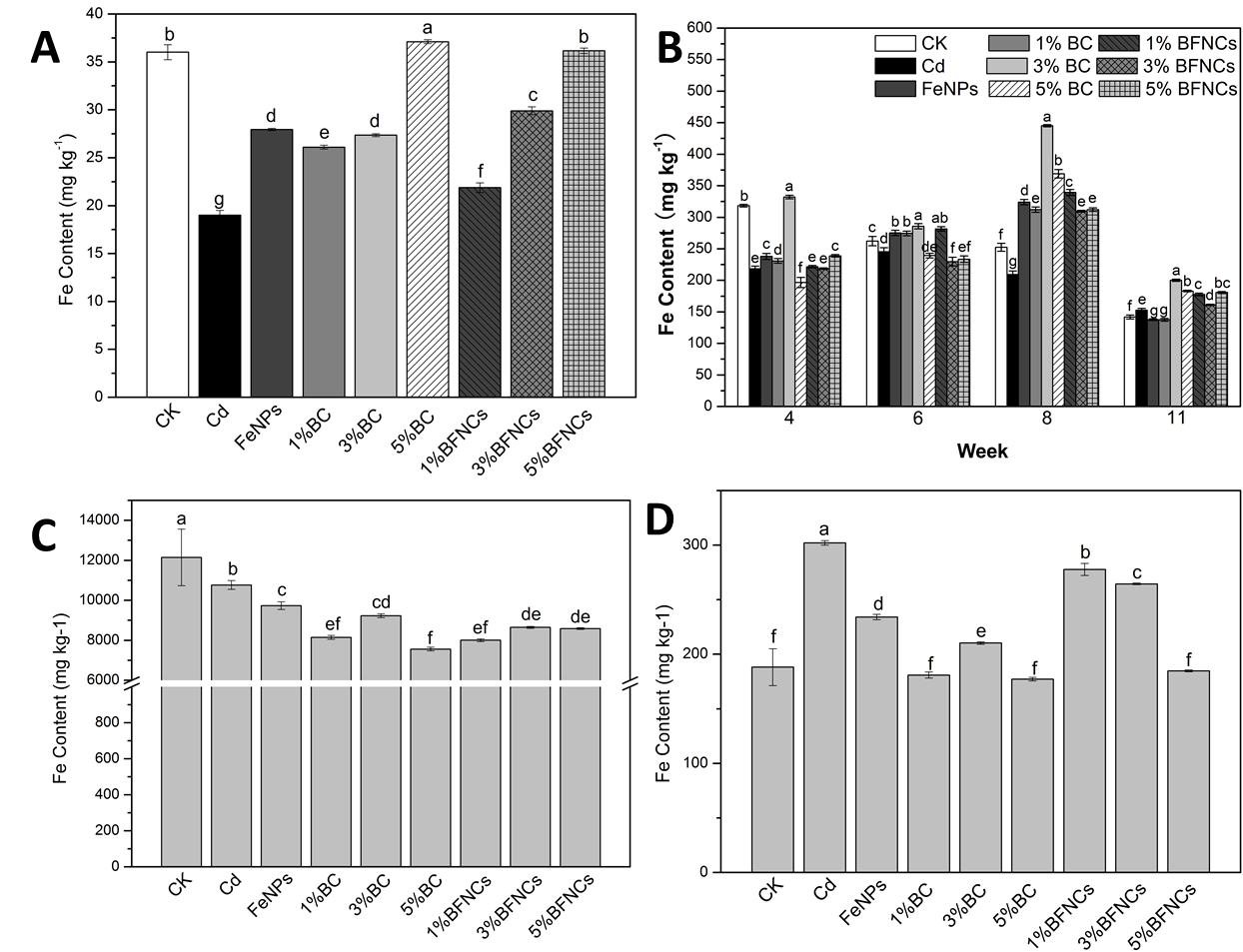
**

**Figure S4.** The content of iron in fruits (A), leaves (B) and total iron (C) and available iron (D) in soil in different treatment groups. The data show the mean ± SD of three replicates. Different lowercase letters indicate significant differences (when p＜0.05).


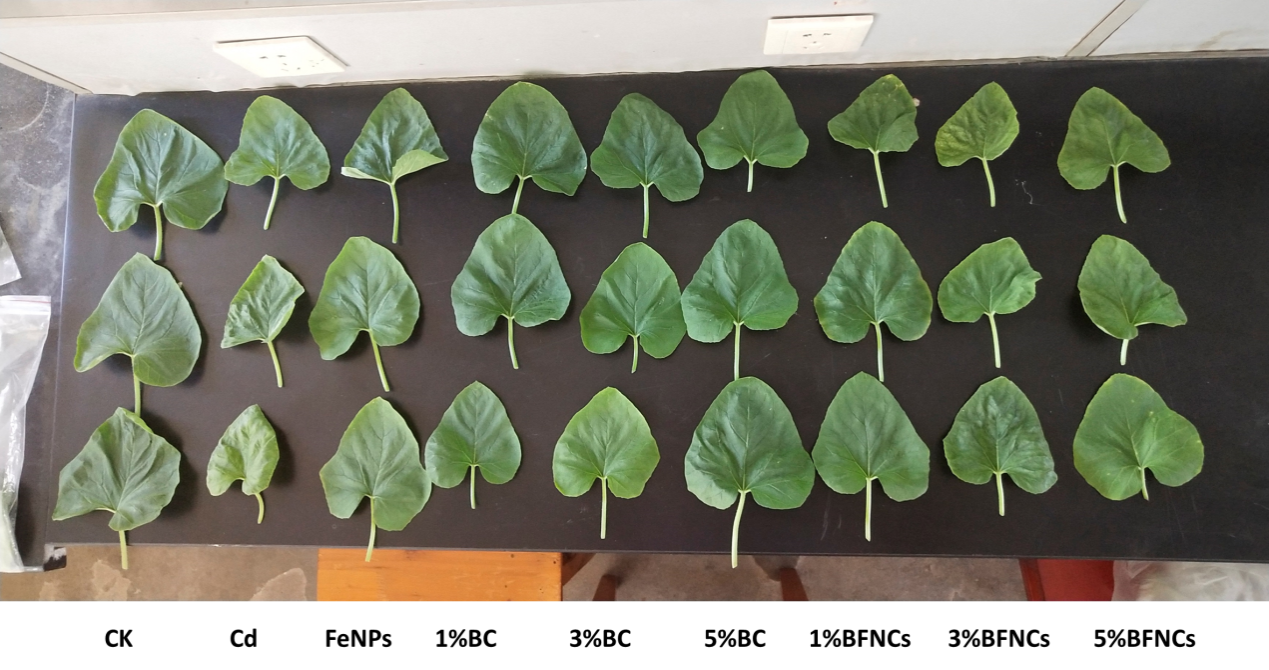


**Figure S5.** Comparison of leaves in different treatment groups of muskmelon plants.

**Table S1.** Physical and chemical properties of soil

| Organic Matter  (g/kg) | Ammonium Nitrogen  (mg/kg) | Nitrate Nitrogen  (mg/kg) | Available Phosphorus  (mg/kg) | Available Potassium  (mg/kg) | Total Fe content  (mg/kg) |
| --- | --- | --- | --- | --- | --- |
| 10.23 | 4.17 | 2.91 | 44.95 | 363.69 | 8960 |
